# An Unusual Case of Pelvic Congestion Syndrome: A Case Report

**DOI:** 10.7759/cureus.75502

**Published:** 2024-12-10

**Authors:** George Mpourazanis, Antonio Simone Laganà, Kostas Tepelenis, Panagiotis Tsirkas, Fani Gkrozou, Minas Paschopoulos, Rüdiger Schulz-Wendtland, Apostolos Ntanasis, Pantelina-Danai Korkontzelou, Ioannis Korkontzelos

**Affiliations:** 1 Department of Obstetrics and Gynecology, General Hospital of Ioannina G. Hatzikosta, Ioannina, GRC; 2 Unit of Gynecologic Oncology, ARNAS "Civico-Di Cristina-Benfratelli" Department of Health Promotion, Mother and Child Care, Internal Medicine and Medical Specialties (PROMISE), University of Palermo, Palermo, ITA; 3 Department of Surgery, General Hospital of Ioannina G. Hatzikosta, Ioannina, GRC; 4 Department of Obstetrics and Gynaecology, University Hospital of Ioannina, Ioannina, GRC; 5 Department of Obstetrics and Gynecology, University of Ioannina, Ioannina, GRC; 6 Department of Gynecology and Obstetrics, University Breast Center for Franconia Erlangen, University Hospital, Friedrich Alexander University of Erlangen-Nuremberg, Erlangen, DEU; 7 Department of Anesthesiology, General Hospital of Ioannina G. Hatzikosta, Ioannina, GRC; 8 Department of Medicine, Medical University of Sofia, Sofia, BGR; 9 Department of Obstetrics and Gynecology, Ioannina State General Hospital “G. Chatzikosta”, Ioannina, GRC

**Keywords:** diagnostic laparoscopy, endometriosis, gnrh analogues, pcs, pelvic congestion syndrome, pelvic pain, pelvic syndrome

## Abstract

This manuscript presents a case of a 35-year-old nulligravida premenopausal woman who presented with acute abdominal pain due to pelvic congestion syndrome (PCS). PCS is characterized by multiple pathophysiological mechanisms and various clinical presentations. Our patient was nulliparous with no history of abdominal surgery. However, diagnostic laparoscopy revealed endometriosis lesions on the uterovesical fold. Reporting this unusual case, we focus on the presenting symptoms, the imaging findings, and the final approach by diagnostic laparoscopy.

## Introduction

Pelvic congestion syndrome (PCS) is a medical condition characterized by complex clinical presentation and is considered one of the underdiagnosed causes of chronic pelvic pain [1]. Worldwide, PCS has an impact on up to a quarter of the female population [2] and is frequently referred to as “pelvic pain syndrome,” “female varicocele,” “pelvic vascular congestion” (PVC), and pelvic venous insufficiency (PVI) [3].

Soysal et al. suggest that as much as 40% of chronic pelvic pain in females may be attributed to PCS [4]. Women with a history of multiple pregnancies are more often affected by PCS, mainly between 20 and 53 years of age [5]. Risk factors for PCS include a high number of pregnancies, structural abnormalities in the pelvic vein, family history of pelvic pain, patient hormonal imbalance such as elevated estrogen levels, conditions like polycystic ovary syndrome, estrogen therapy, varicose veins in the legs, phlebitis, uterine prolapse, prior pelvic surgery, and activities that involve heavy weight lifting or prolonged and frequent standing without relief by walking [6].

The pathogenesis of PCS is multifactorial, with many theories proposed to explain its development. Several studies have identified potential genetic factors linked to PCS, such as mutations in genes FOXC2, TIE2, NOTCH3, and genes involved in the transforming growth factor-β signaling pathway, which may predispose individuals to venous abnormalities and contribute to the development of PCS [7].

Treatment options include embolization, gonadotropin-releasing hormone (GnRH) therapy to suppress ovarian function, progestin hormone therapy to alleviate pain, and hysterectomy. Furthermore, pelvic floor physical therapy can be utilized as an additional therapy, addressing any concurrent pelvic floor dysfunction as well as issues with bowel and bladder function. Behavioral therapy may also be beneficial for managing associated serious conditions such as depression, anxiety, and sexual dysfunction. It is important to note that PCS is mainly diagnosed with the exclusion of other medical conditions [8].

Herein, we report a case of a nulliparous premenopausal woman who presented to the gynecological emergency department complaining of severe abdominal pain in the suprapubic and in the left abdominal area. Transvaginal ultrasonography (TVS) was helpful in order to exclude urgent gynecological pathology. Consecutive diagnostic laparoscopy revealed increased vascularity on the left infundibulopelvic ligament and the pelvic peritoneum and enlarged and prominent veins on the pelvic peritoneum (laparoscopic sign of PCS). Additionally, multiple endometriosis lesions were located mainly on the uterovesical fold.

## Case presentation

A 35-year-old patient was referred to the gynecological emergency department for presenting severe acute abdominal pain in the suprapubic area. The patient was in overall good health and reported symptoms in the past, such as chronic pain during menstrual cycles, diarrhea, constipation, and dysuria. She reported that the pain worsened after sexual intercourse and daily activities such as walking and running. Her vital signs included a blood pressure of 130/98 mmHg, temperature of 36.8°C, pulse rate of 80 beats per minute, respiratory rate of 18 breaths per minute, and oxygen saturation of 98% on room air. Her physical examination was only remarkable for left-sided abdominal pain. Her body mass index (BMI) was calculated to be 18.4 kg/m2. Laboratory results showed no inflammation (Table 1).

**Table 1:** Preoperative and postoperative laboratory assessments AFP: alpha-fetoprotein; ALB: albumin; ALP: alkaline phosphatase; ALT: alanine transaminase; AMY: amylase; AST: aspartate transferase; aPTT: activated partial thromboplastin time; CA 125: cancer antigen 125; CA 15-3: cancer antigen 15-3; CA 19-9: cancer antigen 19-9; CA++: calcium; CEA: carcinoembryonic antigen; CHOL: cholesterol; CRE: creatinine; CRP: C-reactive protein; GGT: gamma-glutamyl transferase; GLC: glucose; HCT: hematocrit; HGB: hemoglobin; INR: international normalized ratio; K+: potassium; LDH: lactate dehydrogenase; Mg++: magnesium; Na+: sodium; PLT: platelet; TBL: total bilirubin; ThCG: thyroid chorionic gonadotropin; TPR: total protein; TRG: triglycerides; UA: uric acid; URE: urea; WBC: white blood cell

Parameter	Day 0 (admission and operation)	Day 1	Day 4 (exit day)	Follow-up six months	Reference number
WBC	10 k/μL	10.5 k/μL	9 k/μL	8 k/μL	4-11 k/μL
Neutrophils	45%	56%	50%	45%	40-75%
HBG	12.5 g/dL	11 g/dL	11.4 g/dL	12 g/dL	11.8-17.8 g/dL
HCT	46%	43%	43.6%	45%	36-52%
INR	1.00	1.00	Not taken	Not taken	0.8-1.2
aPTT	38.36 seconds	30 seconds	Not taken	Not taken	26-36 seconds
PLT	290 k/μL	285 k/μL	286 k/μL	289 k/μL	140-450 k/μL
CRP	0.30 mg/dL	0.40 mg/dL	0.25 mg/dL	0.18 mg/dL	0-0.80 mg/dL
AST	17 U/L	20 U/L	28 U/L	17 U/L	5-33 U/L
ALT	24 IU/L	21 IU/L	25 IU/L	22 IU/L	5-32 IU/L
GGT	15 IU/L	15 IU/L	18 IU/L	27 IU/L	5-31 IU/L
ALP	50 IU/L	53 IU/L	59 IU/L	45 IU/L	35-125 IU/L
AMY	64 IU/L	48 IU/L	50 IU/L	69 IU/L	28-100 IU/L
ALB	4.6 g/dL	4.4 g/dL	4.2 g/dL	3.8 g/dL	3.5-5.1 g/dL
GLC	87 mg/dL	99 mg/dL	90 mg/dL	100 mg/dL	70-115 mg/dL
CHOL	195 mg/dL	192 mg/dL	188 mg/dL	200 mg/dL	120-220 mg/dL
LDH	172 IU/L	211 IU/L	198 IU/L	170 IU/L	120-230 IU/L
TBL	0.59 mg/dL	0.52 mg/dL	0.56 mg/dL	0.60 mg/dL	0.1-1.3 mg/dL
TPR	7.0 g/dL	6.6 g/dL	6.8 g/dL	7 g/dL	6.2-8.4 g/dL
TRG	130 mg/dL	128 mg/dL	110 mg/dL	150 mg/dL	30-160 mg/dL
ThCG	< 2.30 mIU/mL	Not taken	Not taken	Not taken	0-5 mIU/mL
UA	2.9 mg/dL	2.5 mg/dL	2 mg/dL	5 mg/dL	2.3-6.1 mg/dL
URE	19 mg/dL	34 mg/dL	25 mg/dL	40 mg/dL	10-50 mg/dL
CRE	0.86 mg/dL	0.89 mg/dL	0.90 mg/dL	0.95 mg/dL	0.5-1.1 mg/dL
K+	5 mmol/L	4.5 mmol/L	3.8 mmol/L	4.8 mmol/L	3.5-5.1 mmol/L
Na+	145 mmol/L	141 mmol/L	134 mmol/L	140 mmol/L	136-146 mmol/L
Mg++	2 mEq/L	1.89 mEq/L	1.45 mEq/L	1.95 mEq/L	1.3-2.1 mEq/L
CA++	9.4 mg/dL	8.8 mg/dL	8.2 mg/dL	10 mg/dL	8.2-10.5 mg/dL
AFP	<2.00 ng/mL	<1.89 ng/mL	Not taken	<1.50 ng/mL	0.89-8.78 ng/mL
CA 125	22.0 U/mL	18 U/mL	Not taken	14 U/mL	0-35 U/mL
CA 15-3	11.0 U/mL	9 U/mL	Not taken	5 U/mL	0-31.3 U/mL
CA 19-9	40.62 U/mL	38 U/mL	Not taken	25 U/mL	0-37 U/mL
CEA	4.11 ng/mL	3.13 ng/mL	Not taken	2 ng/mL	0-5 ng/mL

**Figure 1:**
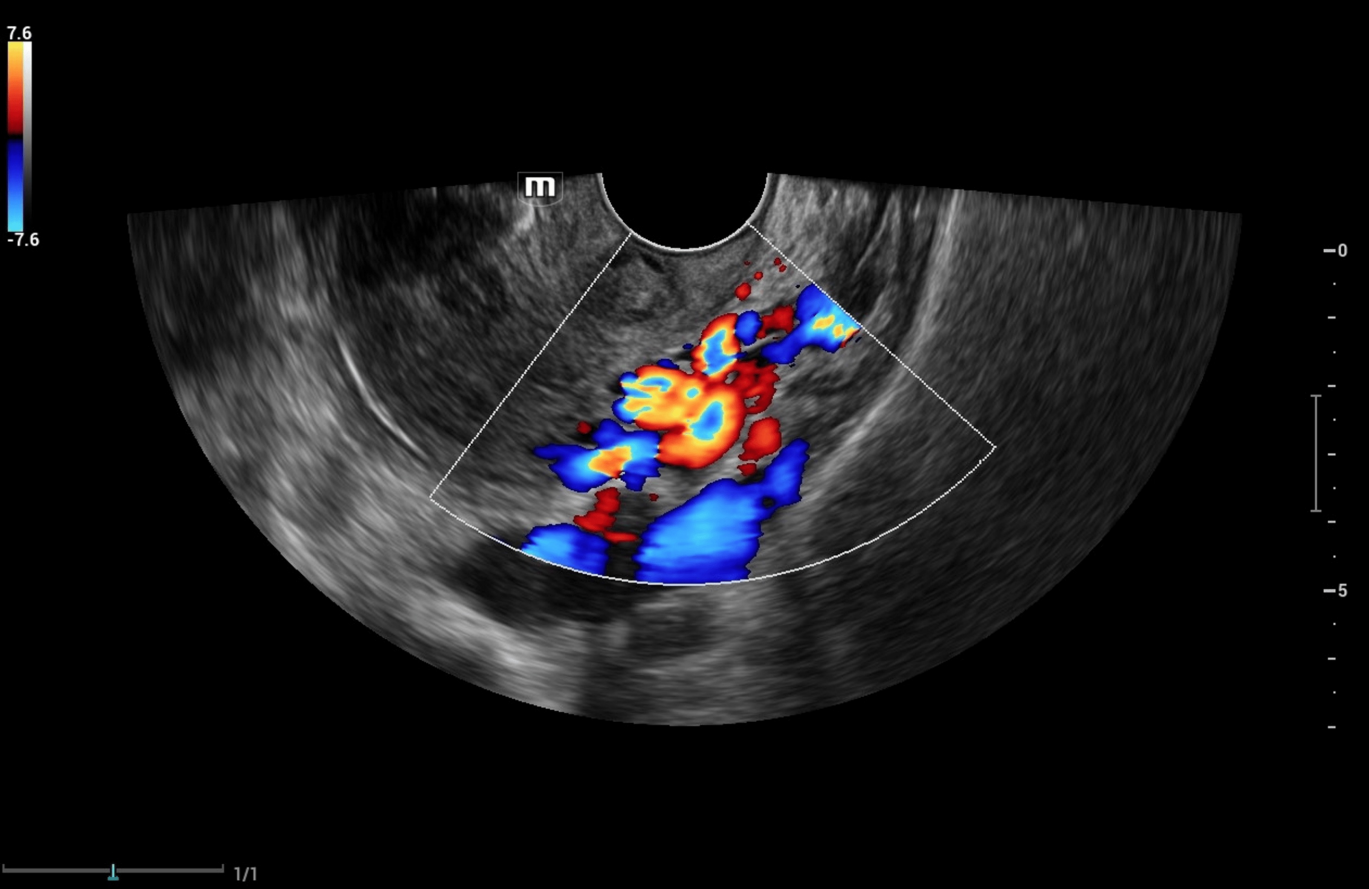
Color Doppler ultrasound shows dilated veins in the adnexae.

A comprehensive medical history revealed that she was nulligravida and had not undergone a gynecological assessment or Pap smear. She had a history of tobacco and alcohol use and did not report any pharmaceutical drug usage. In the past, she had undergone a cystoscopy examination due to her chronic dysuria, which reported a normal urethra and observed external pressure on the urinary bladder on the lateral wall towards the dome without wall changes. TVS ultrasonography revealed a uterus in an anteflexed and anteverted position with normal dimensions, endometrial thickness showing normal findings, and both ovaries within normal limits. On the left side, there were several dilated veins in the adnexa, showing reversed venous flow on color Doppler imaging (Figure 1).

**Figure 2:**
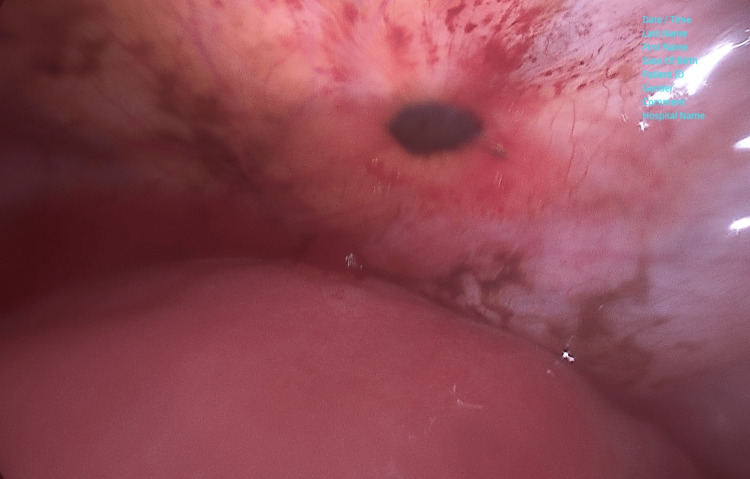
Macroscopic appearance during laparoscopy endometriosis lesions stage I and II at the uterovesical fold.

Due to her allergy history and her severe pain, she mentioned that a CT scan and MRI were contraindicated. After eliminating any other potential underlying condition, with TVS, a PCS was considered. In the operating room, the patient was placed in a lithotomy position and underwent induction of insensibility. A subumbilical incision was performed, followed by the introduction of the Verres pneumoperitoneum. Introduction of two ancillary trocars, left and right, in the lower abdomen. Insertion of uterine manipulator through the vagina. Diagnostic laparoscopy was performed using a 30-degree laparoscopy camera. Laparoscopy findings revealed a normal-sized anteverted uterus, normal mobility of the uterus, and no synechiae identified. Uterovesical fold appeared with multiple endometriosis lesions in stages I and II (Figure 2).

**Figure 3:**
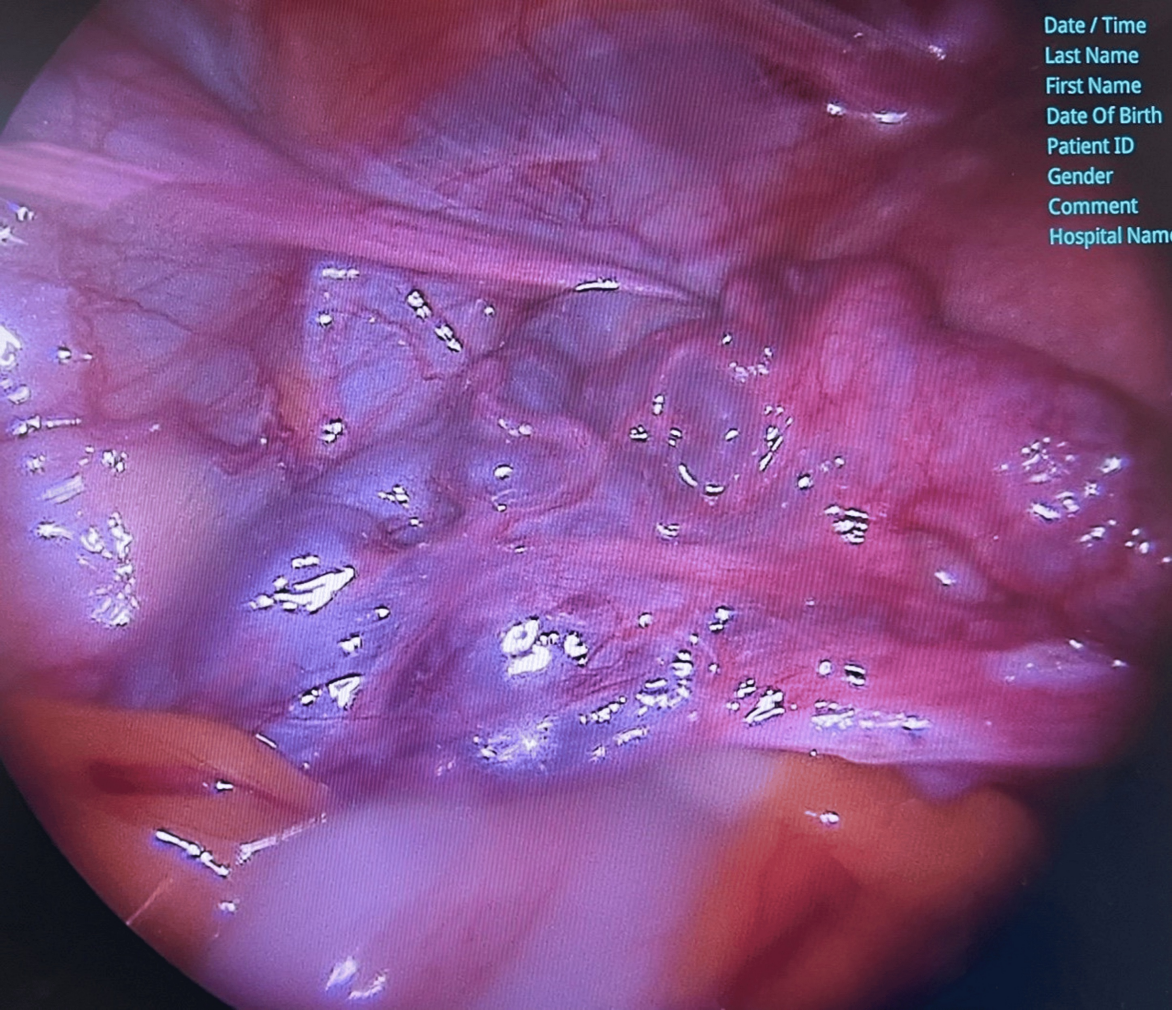
Macroscopic appearance during laparoscopy at the left side of the varicose veins at the left side of the uterus.

Left and right adnexae were normal sizes of ovaries and fallopian tubes. We noticed an increased vascularity on the left infundibulopelvic ligament and pelvic peritoneum with enlarged and prominent veins (laparoscopic sign of PCS) (Figure 3).

Moderate dilation of a small number of veins was identified in the pouch of Douglas. No synechiae were identified in the pelvic area. Laparoscopic inspection of left and right paracolic spaces revealed no obvious pathology. Inspection of the appendix checked normal size and appearance. Left and right diaphragmatic spaces inspected normal. Liver and gallbladder also checked normal. A final diagnosis from the laparoscopy procedure was a PCS, with multiple lesions of endometriosis. We provided the patient with an intramuscular injection of GnRH analogs. At the follow-up appointment at the outpatient clinic of the gynecology department in six months, the patient was symptom-free.

## Discussion

PCS has underlying mechanisms similar to those of male varicoceles, although the specific processes leading to their clinical symptoms remain unclear. Additionally, conditions like May-Thurner syndrome (MTS) show parallels with PCS, particularly in menopausal patients. This syndrome, often seen in older women, occurs when the left iliac vein is compressed by the right iliac vein, increasing the risk of deep vein thrombosis (DVT) or blood clots, which can cause pain along a slightly left trajectory [8].

MTS is frequently confused with PCS [9]. In a study by Siqueira et al., 22 symptomatic patients with PCS aged up to 55 years old were treated with embolization of uterine varices. Notably, the authors indicated that seven patients (31.8%) were postmenopausal at the time of inclusion, although they did not specify how this menopausal status was established. The study found no significant difference in treatment success between premenopausal and postmenopausal patients [10].

A study by Szarflaki et al. investigated the prevalence of ovarian venous congestion in adult patients. Noteworthy, the authors found that 13.7% of a total of 1,042 abdominal and pelvic CT scans performed in females exhibited venous congestion. The average age of the patients in this cohort study was 47 years, suggesting that a significant ratio may have been postmenopausal. However, due to the exploratory nature of the study and its focus on radiological findings, the authors did not specify whether the patients met the clinical criteria for PCS [11].

A systematic review involving 473 patients who underwent interventional coil embolization found that clinical improvement in symptoms occurred in 82.1% up to 100% of the cases. Complications were noted to be infrequent and generally mild, including local hematoma following cannulation. Additionally, the recurrence rate was reported to be very low [12].

It is worth mentioning that these chronic medical conditions, such as PCS, have a strong psychological impact on affected women, like in our case. Studies have shown that chronic pain and discomfort have an increased risk of developing depression, anxiety, and adjustment disorders [13].

Concerning endometriosis, a network meta-analysis of 36 randomized controlled trials showed that GnRH analogs are particularly effective in reducing pelvic pain after six months of treatment. Notably, these analogs were also ranked high for their ability to alleviate dysmenorrhea after three months [14]. In a recent phase III randomized and double-blind study, researchers found that a daily dose of 200 mg of linzagolix, a GnRH antagonist, paired with add-back therapy, significantly reduced both dysmenorrhea and non-menstrual pelvic pain after three months. However, a lower daily dose of 75 mg of linzagolix only showed a significant decrease in dysmenorrhea at the same three-month interval [15].

In the literature, several reports have investigated the complexity (symptoms, diagnosis, and treatment) of PCS. However, this is believed to be the first case of the syndrome presented in a nulliparous woman in association with endometriosis.

## Conclusions

This case highlights the importance of a multidisciplinary approach in managing PCS, especially when compounded by other gynecological conditions like endometriosis. Clinicians should maintain a high level of suspicion for PCS in women with chronic pelvic pain with a thorough evaluation and utilize appropriate diagnostic imaging, endoscopic surgery, and pharmaceutical techniques. PCS typically affects multigravida premenopausal and postmenopausal women, but it can also occur in premenopausal nulligravida women, as demonstrated in this case report. Further research is necessary to understand the precise pathological mechanisms involved and establish a gold-standard treatment method that effectively addresses this syndrome.
